# Acceptance Factors and Psychological Investigation of Clinical Trials in Cancer Patients

**DOI:** 10.1155/2023/5617575

**Published:** 2023-11-24

**Authors:** Jiangjie Sun, Jingyi Fang, Chenchen Zhang, Nannan Jia, Weiming Zhao, Jinjian Gao, Yingying Huang, Jiqing Hao, Liping Zhang

**Affiliations:** ^1^School of Management, Hefei University of Technology, 230039, China; ^2^School of Health Care Management, Anhui Medical University, Hefei, Anhui 230032, China; ^3^Clinical Medical College, Anhui Medical University, Hefei, Anhui 230031, China; ^4^First Clinical Medical College, Anhui Medical University, Hefei, Anhui 230032, China; ^5^First Affiliated Hospital of Anhui Medical University, Hefei, Anhui 230032, China; ^6^School of Marxism, Anhui Medical University, Hefei, Anhui 230032, China

## Abstract

**Aim:**

To understand the degree of oncology patients' awareness of drug clinical trials and oncology patients' willingness to participate in drug clinical trials and the factors influencing them.

**Methods:**

The differences in the relevant variables of patients' willingness to accept clinical trials were analyzed, and a descriptive analysis was done for the measurement data (mean and standard deviation). Pearson's correlation coefficient analysis was used to examine the correlation between willingness and the demographic variables. Stepwise regression analysis was used to explore the influencing factors of patients' willingness to accept clinical trials.

**Results:**

There were no statistical differences in age, gender, education level, marital status, place of residence, monthly income, medical payment method, and treatment time (*P* > 0.05). Patients' willingness to accept drug clinical trials differed in their cognitive degree of clinical drug trials (*P* = 0.002). Patients' willingness to accept drug clinical trials differed in their experience in clinical trials (*P* < 0.001). The correlation difference was statistically significant. The willingness to accept drug clinical trials was negatively correlated with treatment time (*R* = −0.16, *P* < 0.05) and positively correlated with awareness of clinical trials and whether they had been subjects (*R* = 0.16 and 0.43, *P* < 0.05). Multiple regression analysis showed that patients' willingness was directly influenced by age, treatment time, and whether they had been subjects (*F* = 21.315, *P* < 0.001).

**Conclusion:**

Age, treatment time, and whether they had been subjects were the direct influencing factors of patients' willingness. This study pointed out that hospitals should do a good job in the publicity of clinical trials of new drugs, expand publicity channels, increase publicity efforts, improve the awareness of clinical trials of the masses, and promote the enthusiasm of the masses to participate in clinical trials of drugs.

## 1. Introduction

In the 21st century, cancer has become the main killer that threatens human health and longevity. According to the latest global cancer statistics report published by the International Agency for Research on Cancer (IARC) in the Journal of Clinician Cancer in 2021, the estimated number of new cancer cases in the world in 2020 is 19 292 789. The age-standardized incidence rate by world standard population (ASIRW) was 2,010 per million, which was significantly higher than that in 2018 (1,979 per million) [1]. The trial of new cancer drugs is the key to the clinical application of new drugs, and it also provides opportunities and possibilities for cancer patients to improve their quality of life and prolong their lifespan.

### 1.1. Current Status of Cancer

According to statistics from the World Health Organization in 2019, cancer is currently the first or second leading cause of death in 112 countries [2]. Cancer not only afflicts patients physically but also ravages their mental health. Zabora et al. investigated the psychological status of 4,496 cancer patients and found that the proportion of patients with depression and anxiety was as high as 35.1% [3]. The worse the prognosis and the heavier the burden of the disease, the higher the degree of depression and anxiety. On the other hand, cancer also brings a heavy burden to the society and economy. As far as China is concerned, China has a population of 1.4 billion. Even a slight increase in the incidence and mortality of a certain type of cancer will affect the life expectancy of a sufficient number of people and consume a large number of social medical resources [4]. Therefore, in this fierce war between all mankind and cancer, scientists must race against time to research therapeutic drugs and seek the well-being of human beings for a healthy life.

### 1.2. Comparison of Current Situation of Drug Clinical Trials at Home and Abroad

The research and development of new drugs is not easy. As a crucial step from laboratory research to clinical application, drug clinical trials naturally play a pivotal role. China once hindered drug innovation due to a long drug review process and strict clinical trial application policies [5]. In order to encourage drug innovation, in 2015, the State Council of the People's Republic of China issued a landmark policy—“Opinions on Reforming the Approval System for Drugs and Medical Devices” [6], which accelerated the new process of domestic anticancer drug research and development. As of April 2020, there were a total of 1,974 drug clinical trial institutions recognized by the State Drug Administration, including 888 antitumor drug clinical trial institutions [7]. However, the National Cancer Institute's Clinical Trials Collaborative Program includes 3,100 institutions and 14,000 investigators [8]. Based on the differences in the population base and national conditions of cancer between China and the United States, China's current drug clinical trial research and development is still far from meeting the needs of China's drug research and development. Recruiting a sufficient number of subjects for clinical data research and confirming the efficacy and safety of new drugs are the key to successful clinical trials of new drugs. However, in China, patients' cognition and participation in clinical trials are not high. Zhang investigated the acceptance of clinical trials in 678 cancer patients, and only 42.1% of patients expressed willingness to participate in clinical trials [9], which is much lower than that of foreign patients whose results are 50%-80% [10, 11]. In a comparative survey of urban and rural patients' attitudes toward clinical research in the United States and China, it was found that compared with Chinese patients, American patients may be less concerned about participating in research [12]. In order to increase the participation of domestic patients in clinical trials and accelerate the process of new drug research and development, we need to understand the willingness and motivation to participate in clinical trials of new drugs.

### 1.3. Factors Related to the Willingness of Patients to Participate in Clinical Trials

We noted that in the study by Cao et al. [13], people who know about drug clinical trials were more inclined to participate in the trial. Age, gender, financial income, and the level of concern of health care professionals were found to be relevant factors influencing oncology patients' participation in clinical trials in the study by Zhang [9], and the study by Huang et al. and Lang et al. showed that physicians' concern was also an important factor influencing patients' choices [14, 15]. In 2016, Igwe et al. used the Attitudes on Randomized Trials Questionnaire to study the attitudes and willingness of American patients to participate in clinical trials [16] and concluded that psychological stress is not an important factor affecting patients' participation in clinical trials. However, considering the differences in public cognition between China and the United States, we designed a questionnaire and added new variables that might affect the willingness of patients to accept clinical trials on the basis of previous studies to carry out investigations and studies.

Based on the research literature and findings of previous researchers, we propose the following hypotheses:

**Hypothesis 1 .**

There are demographic differences in patients' willingness to participate in drug clinical trials.

**Hypothesis 2 .**

There is a correlation between patients' willingness to participate in drug clinical trials and their experience in clinical trials.

**Hypothesis 3 .**

Patients' willingness to participate in drug clinical trials is related to their mental health factors.

From June to December 2021, this study conducted a survey on the willingness and psychological factors of cancer patients to accept clinical trials in the Department of Oncology, the First Affiliated Hospital of Anhui Medical University, and collected a total of 211 survey results from oncology patients.

## 2. Method

### 2.1. Data Collection

The First Affiliated Hospital of Anhui Medical University is located in Hefei, the capital city of Anhui Province, with a superior geographical position, and its hospital has been shortlisted in the list of China's top 100 hospitals for many consecutive years and ranks first in Anhui Province, with strong comprehensive strength. In addition, the Department of Oncology, as a key department of the First Affiliated Hospital of Anhui Medical University, receives and treats cancer patients from all over the province, with a rich sample size. Therefore, the Department of Oncology of the First Affiliated Hospital of Anhui Medical University is a very suitable place for this questionnaire survey.

A total of 220 questionnaires were distributed, and 211 were recovered, with an effective rate of 95.9%. Of the valid responses, 109 were from men, and 102 were from women. There were 4 cases aged 30 years and younger, 58 cases aged 31 to 50 years old, 106 cases aged 51 to 70 years old, and 43 cases over 70 years old. Other demographic characteristics are detailed in Table 1.

The survey was conducted by clinical medical undergraduates trained by medical professionals. Before the survey, the reference answer points were stipulated, the scoring criteria were unified, and the possible answers were predicted. If the patient has doubts, the investigator could help him explain the meaning of the question but must not inspire, induce, or add subjective will, and the answer truly reflected the situation of the respondent; the questionnaire was withdrawn on the spot after answering the questionnaire.

Due to the large number of respondents, the authors of this study signed a document promising to obtain the oral informed consent of all respondents to the survey. This was approved by the Ethics Committee of Anhui Medical University.

### 2.2. Research Tools

The self-compiled questionnaire was divided into three parts; the first part was to collect demographic data of the respondents, mainly including the patient's gender, age, educational level, marital status, place of residence, monthly income, medical payment methods, medical information, satisfaction with treatment, and treatment time.

The second part was to investigate the patient's awareness of clinical trials, willingness, and the reasons for their participation or rejection. A total of 7 small problems in 3 aspects were designed, and some problem options were also assigned to deal with. These are as follows: The first survey was to investigate whether patients were aware of the trial and related knowledge (yes (=1) and no (=2)), and if patients chose yes, they were further asked how they learned about it: doctor, relative or friend, or media such as books or the internet. The second survey investigated whether respondents had participated in drug clinical trials as subjects (yes (=1) and no (=2)). The third item asked the respondents about their willingness after the researchers informed the basic operation of the clinical trial in detail (willing to participate (=1), undecided (=2), and unwilling to participate (=3)). We further asked why they had joined or refused, and who they were turning to for help. The results of the analysis are shown below.

The third part was to use the self-rating anxiety and depression scale to understand the mental health of patients. The self-rating anxiety scale is a tool for measuring anxiety developed by Zung in 1971 [17]. The test is a short-distance self-assessment scale, easy to operate, time-consuming, and not affected by factors such as age, gender, and economic status; the scope of application is quite wide, and it is also one of the common tools used in psychiatric clinics. Tian et al. used the self-rating anxiety scale for clinical verification, which proved that it has good reliability and validity and can be used for clinical application (Cronbach's coefficient is 0.897, *P* < 0.001) [18]. Therefore, SAS was used in this study as a tool to assess the degree of anxiety in cancer patients. The self-rating depression scale is a tool developed by Zung in 1965 to measure depression [19, 20]. Li et al. used the self-rating depression scale for clinical validation, demonstrating that it has good reliability and validity and can be used for clinical application (Cronbach's coefficient 0.92, *P* < 0.001) [21].

### 2.3. Statistical Methods

Two members of the research team reviewed all the questionnaire data and used EpiData 3.1 to enter the data. Descriptive analysis of the measured data was performed using SPSS 19.0 [22]. This article conducted a difference analysis of relevant variables related to patients' willingness to accept clinical trials; a descriptive analysis was done for the measurement data (mean and standard deviation). Pearson's correlation coefficient analysis was used to examine the correlation between willingness and the demographic variables. Stepwise regression analysis was used to explore the influencing factors of patients' willingness to accept clinical trials. When *P* < 0.05, it was considered statistically significant.

## 3. Results

### 3.1. Differences in General Demographic Information

Based on the preliminary statistics of demographic variables and cognitive status, the results of comparing differences are shown in Table 1. 54.0% of the respondents had a junior high school education or less, 89.1% of the respondents were married, 57.8% lived in rural areas, and 42.2% had a monthly income of less than 1,000 yuan. Those between 1,000 and 5,000 yuan accounted for 50.7%, and nearly all (94.8%) of the patients were covered by medical insurance. The stratified proportion of treatment time was evenly distributed, accounting for 21.8% less than three months, 37.4% from three months to one year, 27.0% from one year to three years, and 13.7% for more than three years. 70.2% of the respondents did not pay attention to medical information. Of the 211 patients, based on their completed SAS and SDS scale scores, 112 had no anxiety, 61 had mild anxiety, 33 had moderate anxiety, and 5 had severe anxiety. 120 had no depression, 73 had mild depression, 10 had moderate depression, and 8 had severe depression; details are shown in Table 1.

We compared the basic information of patients in the three groups who chose to participate in the clinical trial with those who were unwilling to participate and those who could not make up their minds. The results showed that there was no statistical difference in age, gender, education level, marital status, place of residence, monthly income, medical payment method, and treatment time (*P* > 0.05), which denied Hypothesis 1; There were differences in patients' willingness to accept drug clinical trials with respect to their awareness of clinical drug trials (*P* = 0.002) and differences in patients' willingness to accept drug clinical trials with respect to their historical experience in clinical trials (*P* < 0.001), and the relevant differences were statistically significant, which laid the foundation for the study of factors influencing patients' willingness to accept drug clinical trials.

Based on sample data of patients' willingness, SAS, SDS, and demographic variables, we create a binary variable correlation matrix (Table 2).

It can be concluded from Table 2 that the patients' willingness to accept drug clinical trials was negatively correlated with treatment time and positively correlated with their awareness of clinical trials and whether they had been subjects, which verified Hypothesis 2. There was no statistically significant correlation between patient' s willingness to accept drug clinical trials and SAS and SDS scores, which initially denied Hypothesis 3. Treatment time was negatively correlated with patients' age. Awareness of clinical trials was negatively correlated with education level and treatment time; whether they had been subjects was negatively correlated with payment method and positively correlated with awareness. SAS score was positively correlated with age. SDS score was positively correlated with the SAS score and negatively correlated with gender, and marital status was positively correlated with age. Place of residence was negatively correlated with education level, monthly income was positively correlated with education level, and monthly income was negatively correlated with place of residence. The ways to know the knowledge of clinical trials were negatively correlated with the place of residence and positively correlated with whether they had been subjects.

According to the analysis results in Table 2, variables directly and indirectly related to patients' willingness were selected for stepwise multiple regression analysis [23, 24], and the results are shown in Table 3.

The results of multiple regression analysis showed that age, treatment time, and whether they had been subjects were the direct influencing factors of patients' willingness, while the remaining related variables may be the indirect influencing factors.

## 4. Discussion

There were no statistical differences in patients' willingness to accept drug clinical trials with respect to age, gender, education level, marital status, place of residence, monthly income, and medical payment method as well as treatment time. The possible reasons for this were due to insufficient access to information on clinical trials, and as shown by the information of 211 oncology patients in this survey, only 64 patients were aware of clinical trial programs and related knowledge. A survey of 64 patients found that 40 (62.5%) were referred by their doctors, 11 (5.2%) by friends and relatives, and 13 (6.2%) through media channels such as books and the Internet. The second reason may be patients' lack of trust in doctors. For example, according to the survey results, the top three reasons for unwillingness to participate in clinical trials were fear of adverse reactions of new drugs (61.9%), fear of delaying the routine treatment (44.0%), and unwillingness to be treated as experimental subjects (32.1%). This was consistent with Sun et al.'s findings that doctor-patient trust is low [22]. This was also the reason why patients' willingness to accept drug clinical trials had significant differences in their cognitive degree of clinical drug trials. Of course, other reasons cannot be ruled out. The reasons for the differences in patients' willingness to accept drug clinical trials with respect to their historical experience in clinical trials were that historical experience increased patients' knowledge of clinical trials, their experience of free treatment in clinical trials, or their trust in doctors due to their better experience in previous clinical trials, which was consistent with the results of this study that “the top three reasons for willingness to participate was to try new treatment drugs (68.5%), trust in doctors and team (65.8%) and to get free treatment (47.9%)”.

Patients' willingness to accept clinical trials of drugs was negatively correlated with treatment time and positively correlated with awareness of clinical trials and whether they had been subjects. One of the possible reasons for this is that patients who have been cured for too long have lost confidence in their own health and trust in their doctors, leading to a decrease in willingness to accept clinical trials. Patients with higher cognitive level and more clinical trial historical experience had a higher understanding of patient drug clinical trials, so it is understandable that they had a higher level of support for clinical trials. This further reflects the low awareness rate and lack of publicity of clinical trials in China, which is consistent with the results of this survey. Out of 211 cancer patients surveyed, only 64 (30.3 percent) said they had heard of and understood the concept of clinical trials, much lower than the 76.5% of Korean cancer patients [18]. At the same time, we also conducted a survey on patients who knew clinical trial knowledge and found that 62.5% of them obtained information from doctors' introduction, while only 6.2% of them learned information from books, the Internet, and other media. However, the situation in South Korea was different from ours [21]. 37.5% of patients in South Korea collected information from doctors or nurses, and 34.3% got information from mass media including TV, newspapers, and the Internet. It can be seen that the lack of appeal and publicity of the domestic medical media for clinical trials fails to let the general public get relevant information and makes it difficult to recruit volunteers for clinical trials in China. If only relying on doctors and other staff to spread the trial information, it is quite difficult; therefore, both medical websites and medical newspapers and magazines should make efforts to promote drug clinical trial projects. If only everyone knows, anyone will participate. The second reason is that the more knowledgeable one is about clinical trials, the more likely one is to choose to participate in a trial study. Those who had done the trial were more willing to participate in the trial than those who had not done it, which may be related to the fact that those who had experienced the trial process were more knowledgeable about it and its operation and trusted drug clinical trials more. This is consistent with the findings of Huang et al. and Lang et al. [14, 15]. Therefore, it is not only the responsibility of medical staff but also the obligation of the whole society to popularize the relevant knowledge of drug clinical trials for patients. Only by promoting the popularization of knowledge related to clinical trials can we improve the enthusiasm of patients to participate in the development of new drugs in China and benefit more cancer patients. However, there is a lack of attention to clinical trial knowledge and volunteer recruitment in China. Just relying on a small board in front of the hospital or a small corner of the publicity board is not enough to achieve the purpose of publicity. Although recruitment information in the Internet era will be published on the official website of the hospital or commercial recruitment website, this information is fragmentary, and the website itself lacks attention. Therefore, it is imperative to establish authoritative and popular clinical trial recruitment websites.

The possible reason for the negative correlation between treatment time and age of patients is that the older the patients are, the weaker their physical function is, the more difficult it is to recover, and the longer the treatment time is. Awareness of clinical trials was negatively correlated with education level and treatment time, which may be because the higher the education level, the stronger the ability to accept new knowledge, and the more likely to worry. The longer the treatment time, the greater the family economic expenditure, the weaker their own health confidence, and the worse the trust between doctors and patients, so there was a negative correlation. This may also be the reason why subjects' experience was negatively correlated with payment method and positively correlated with awareness of clinical trials. The results of the current survey show the reason for the low degree of willingness of patients to participate. The results of this survey showed that only 34.6% of the patients were willing to participate in clinical trials, which is a relatively low willingness to participate compared to both domestic (40.9%-93.3%) and foreign (56.7%-88.0%) studies [13–15, 25–28]. The reasons for this may be as follows: First, in this study, unlike the previous scale design, we added the option of “uncertainty” to the choice of whether the patients were willing to participate in the clinical trial. Secondly, the respondents were not well informed about the clinical trial, and although the author has explained it in detail, the patients could not accept the new and unfamiliar thing in a short period of time. Through further analysis, it was found that 68.5% of the patients wanted to try new drugs, and 47.9% of the patients wanted to get free treatment, which were the main driving forces for the patients to participate in the trial, while only 16.4% of the patients wanted to make contributions to the medical cause. Different from the Chinese who take self-interest as the motivation for participation, altruism and promoting scientific development are more important motivations in American patients [12]. The main reasons for Chinese patients' unwillingness to participate were fear of adverse drug reactions (61.9%) and fear of the impact of interruption of routine treatment (44.0%). Chinese patients pay more attention to safety, while American patients have greater concerns about the privacy of participating in clinical research, which is also the main reason for their reluctance to participate in clinical research [12]. In the question “Who would you turn to if you couldn't make up your mind?” 68.5% of the patients chose to ask the doctor's opinion, which was consistent with 65.8% of the patients who chose “trust to the doctor” as the reason for participating in the experiment. Therefore, physicians play an important role in promoting patient participation in clinical trials. Different from previous studies on factors affecting the willingness of patients to participate in clinical trials, we took the psychological status of patients into consideration, but the results showed that the mental health status of patients did not affect the willingness of patients to participate in clinical trials. This result was consistent with the results of Igwe et al.'s study in the United States [16]. Meanwhile, we found that patients with clinical trial experience had higher levels of depression, which may be worthy of further attention, and lest depression affect patients' decision of clinical trial intention.

The results of regression analysis showed that age, treatment time, and whether they had been subjects were the direct influencing factors of patients' willingness. The possible reasons for this are that age is directly related to patients' physical function, which determines patients' recovery and healing time, which in turn affects patients' level of trust in physicians and directly influences patients' willingness to conduct clinical trials; patients' clinical trial experience determines patients' sense of clinical trial experience and directly determines patients' decision to conduct clinical trials again.

## 5. Conclusion

The results of this study indicate that age, treatment time, and whether they had been subjects are the direct influencing factors of willingness. Domestic drug clinical trial centers should reasonably analyze the age characteristics of patients, timely carry out the knowledge propaganda of new drug clinical trials for patients, play the value of advocacy of patients with clinical trial historical experience, address the low degree of understanding of clinical trials and the limitation of knowledge channels for tumor patients, actively do a good job of propaganda, expand propaganda channels, and increase propaganda efforts, which not only help to improve the public's knowledge of clinical trials but also promote the enthusiasm of the public to participate in clinical trials. It will not only promote the enthusiasm of people to participate in drug clinical trials but also accelerate the development of tumor-related drug therapy in China.

## 6. Limitations

There are few survey samples. Compared with more than 500 samples investigated in other studies, this study is slightly insufficient. According to the criteria proposed by Comrey, the size of 100 samples is too small, while 200 are qualified, 300 are excellent, 500 are good, and 1000 are very good [29, 30]. For the general analysis of the following 40 project factors, 200 samples are sufficient in most cases. The investigation unit is limited to the oncology department of the First Affiliated Hospital of Anhui Medical University, which lacks national representation. In the next step, we can expand the scope of the research area and the number of research samples. Due to the limitation of the actual survey environment, this study adopts convenient sampling rather than random sampling. Besides, we do not take into consideration other factors (e.g., stage and severity of disease and personality traits) that may influence participants' choice to join clinical trials.

## Figures and Tables

**Table 1:** Demographic characteristic variables and sample distribution.

Variables	Total (*N* = 211)	Likely (*N* = 73)	Not likely (*N* = 54)	Undecided (*N* = 84)	*χ* ^2^	*P*
Age					5.343	0.254
≤50	62 (29.4%)	22 (30.1%)	20 (37.1%)	20 (23.8%)		
51-70	106 (50.2%)	34 (46.6%)	28 (51.9%)	44 (52.4%)		
>70	43 (20.4%)	17 (23.3%)	6 (11.1%)	20 (23.8%)		
Gender					2.494	0.287
Male	109 (51.7%)	41 (56.2%)	23 (42.6%)	45 (53.6%)		
Female	102 (48.3%)	32 (43.8%)	31 (57.4%)	39 (46.4%)		
Education					5.827	0.437
Uneducated	61 (28.9%)	18 (24.7%)	12 (22.2%)	31 (36.9%)		
Junior high school and below	114 (54.0%)	40 (54.8%)	34 (63.0%)	40 (47.6%)		
High school and junior college	30 (14.2%)	13 (17.8%)	7 (13.0%)	10 (11.9%)		
Undergraduate and above	6 (2.8%)	2 (2.7%)	1 (1.9%)	3 (3.6%)		
Marital status					2.640	0.640
Married	188 (89.1%)	68 (93.2%)	48 (88.9%)	72 (85.7%)		
Divorced & Unmarried	10 (4.7%)	2 (2.8%)	2 (3.7%)	6 (7.2%)		
Widowed	13 (6.2%)	3 (4.1%)	4 (7.4%)	6 (7.1%)		
Residence					2.786	0.594
Urban	40 (19.0%)	10 (13.7%)	13 (24.1%)	17 (20.2%)		
Town	49 (23.2%)	19 (26.0%)	10 (18.5%)	20 (23.8%)		
Countryside	122 (57.8%)	44 (60.3%)	31 (57.4%)	47 (56.0%)		
Monthly income					3.956	0.413
<1000	89 (42.2%)	27 (37.0%)	27 (50.0%)	35 (41.7%)		
1000-5000	107 (50.7%)	38 (52.1%)	24 (44.4%)	45 (53.6%)		
>5000	15 (6.2%)	8 (10.9%)	3 (5.6%)	4 (4.8%)		
Medical payment					0.352	0.838
Medical insurance	200 (94.8%)	69 (94.5%)	52 (96.3%)	79 (94.0%)		
At own expense	11 (5.2%)	4 (5.5%)	2 (3.7%)	5 (6.0%)		
Treatment time					12.501	0.052
<3 months	46(21.8%)	8(11.0%)	15(27.8%)	23 (27.4%)		
Three months to one year	79 (37.4%)	26 (35.6%)	24 (44.4%)	29 (34.5%)		
One to three years	57 (27.0%)	26 (35.6%)	9 (16.7%)	22 (26.2%)		
>3 years	29 (13.7%)	13 (17.8%)	6 (11.1%)	10 (11.9%)		
Awareness					12.339	0.002
Yes	64 (30.3%)	33 (45.2%)	10 (18.5%)	21 (25.0%)		
No	147 (68.7%)	40 (54.8%)	44 (81.5%)	63 (75.0%)		
Subject					53.321	<0.001
Yes	30 (14.2%)	28 (38.4%)	0 (0.0%)	2 (2.4%)		
No	181 (85.8%)	45 (61.6%)	54 (100.0%)	82 (97.6%)		

Notes: education, level of education received; marriage, marital status; residence, place of residence; medical payment, medical payment method; treatment time, the duration of the patient's treatment; awareness, patient's awareness of the clinical trial; subjects, patient's historical experience in clinical trials (the results were obtained through appropriate statistical processing herein).

**Table 2:** Correlation matrix of willingness and demographics and SAS and SDS scoring variables.

	Mean	SD	1	2	3	4	5	6	7	8	9	10	11	12	13	14
(1) Age	58.60	12.95	1													
(2) Gender	1.48	0.50	**-0.33**	1												
(3) Education	1.91	0.73	**-0.18**	-0.11	1											
(4) Marriage	2.10	0.53	**0.14**	-0.07	-0.06	1										
(5) Residence	2.39	0.79	-0.03	0.11	**-0.39**	-0.01	1									
(6) Monthly income	1.66	0.64	-0.08	-0.12	**0.42**	0.02	**-0.27**	1								
(7) Payment method	1.05	0.22	-0.02	-0.06	-0.06	0.07	0.10	-0.01	1							
(8) Treatment time	2.33	0.97	**-0.14**	0.04	0.10	-0.05	0.01	0.00	0.01	1						
(9) Awareness	1.71	0.47	0.05	-0.02	**-0.18**	0.01	0.07	-0.05	-0.13	**-0.18**	1					
(10) Subject	1.86	0.35	-0.05	0.07	0.06	0.05	-0.13	0.04	**-0.15**	-0.13	**0.53**	1				
(11) Ways	1.58	0.81	0.05	-0.23	0.24	-0.09	**-0.35**	0.06	-0.03	-0.16	0.07	**0.46**	1			
(12) SAS	45.33	13.01	**0.15**	0.07	-0.07	-0.09	0.02	0.01	0.00	-0.06	**0.16**	0.05	0.17	1		
(13) SDS	50.69	11.80	0.04	**0.19**	-0.07	-0.06	0.04	-0.08	-0.04	-0.02	**0.26**	**0.25**	0.20	**0.62**	1	
(14) Willingness	2.05	0.86	0.00	0.02	-0.10	0.03	-0.06	-0.09	0.01	**-0.16**	**0.16**	**0.43**	0.24	0.01	0.10	1

Note: The correlations marked in bold in the table are statistically significant. Ways are patients' access to clinical trials. The connotation of other variables is shown in Table 1.

**Table 3:** Prediction of willingness.

Predictor	Step 1		Step 2		Step 3	
	B/Coef	SE	B/Coef	SE	B/Coef	SE
Willingness						
Age	-.002	.006	-.008^∗^	.004	-.008^∗^	.004
Gender	.029	.165				
Education	.038	.131	-.084	.067		
Marriage	-.010	.149				
Residence	-.024	.108				
Monthly income	-.152	.124				
Payment method	.126	.277	.089	.214		
Treatment time	-.255^∗∗^	.077	-.134^∗∗^	.050	-.133^∗∗^	.049
Awareness	-.205	.323	-.128	.123		
Subject	1.085^∗∗∗^	.184	1.030^∗∗∗^	.162	.921^∗∗∗^	.136
Ways	.080	.106				
SAS	-.005	.007				
SDS	-.002	.009				
Adjust *R*^2^	.491		.223		.225	
*F*	5.676^∗∗∗^		11.042^∗∗∗^		21.315^∗∗∗^	

Notes: ^∗^, ^∗∗^, and ^∗∗∗^ indicate *P* < 0.05, *P* < 0.01, and *P* < 0.001.

## Data Availability

The data used to support the findings of this study are included in the article.
